# Prevalence and risk factors for chronic endometritis in patients with adenomyosis and infertility: a retrospective cohort study

**DOI:** 10.1186/s12905-024-03245-2

**Published:** 2024-07-16

**Authors:** Jingjing Li, Jiajia Wei, Saiqiong Chen, Xindan Wang, Jing Chen, Dingyuan Zeng, Li Fan

**Affiliations:** Department of Gynecology, Liuzhou Maternity and Child Healthcare Hospital, No. 50, Boyuan Avenue, Yufeng District, Liuzhou City, Guangxi China

**Keywords:** Adenomyosis, CD138, Chronic endometritis, Hysteroscopy, Infertility

## Abstract

**Background:**

To explore the incidence of chronic endometritis (CE) in patients with infertility and different forms of adenomyosis and analyze potential high-risk factors for infection.

**Methods:**

This retrospective cohort study included 154 patients with infertility in the Liuzhou Maternity and Child Healthcare Hospital. Among them, 77 patients with adenomyosis were divided into four subgroups based on magnetic resonance imaging (MRI): internal, exterior, intramural, and full-thickness. Meanwhile, 77 patients did not have adenomyosis. Hysteroscopy and endometrial biopsy were performed in the proliferative phase. The main outcome measures were the morphology of the endometrium, syndecan-1 (CD138) immunohistochemical staining, clinical characteristics, and prevalence of CE in the adenomyosis subgroups.

**Results:**

In comparison to the non-adenomyosis group, the adenomyosis group had significantly higher body mass index (BMI) and CA125 levels. The menstrual cycle in the adenomyosis group was significantly shorter, and menarche was significantly earlier. In comparison to the non-adenomyosis group, the adenomyosis group had a significantly higher diagnostic rate of CE (75.3% vs. 46.8% according to hysteroscopy and 74.0% vs. 33.8% according to histopathology, both with *p* < .050). The incidence of CE was significantly lower in patients with internal adenomyosis when compared with the other three subgroups. Increased BMI contributed to a higher risk of CE.

**Conclusions:**

The prevalence of CE was significantly higher in patients with adenomyosis and infertility. The differences in the incidence of CE are closely associated with the classification of adenomyosis. When patients with infertility are diagnosed with adenomyosis, it is recommended to identify the subtype and screen for endometritis.

## Abstract (summary)

The prevalence of CE in patients with adenomyosis and infertility is significantly higher and differences in the incidence of CE are closely associated with the classification of adenomyosis.

## Background

Chronic endometritis (CE) is a type of endometrial inflammation characterized by plasma cell incursion into the endometrial matrix region [1]. Although CE is frequently silent or presents as non-specific clinical symptoms such as abnormal uterine bleeding, pelvic discomfort, leukorrhea, and minor gastrointestinal discomfort, it may cause decreased fertility and impede the implantation of embryos [2–5]. Women who are infertile and have a history of endometriosis, recurrent pregnancy loss (RPL), and repeated implantation failure (RIF) are at a higher risk of developing CE. Recently, the incidence rate of CE is 14–42% in cases of RIF and 27–57.8% in cases of RPL [6]. Traditionally, CE is diagnosed by endometrial sample histopathology analysis, hysteroscopy, and microbiological culture. Plasma cell identification through histological analysis of endometrial biopsies is the gold standard for diagnosis [7, 8]. Emerging evidence suggest that no fewer than five plasma cells for each high-power field (HPF) could accurately characterize a CE diagnosis with important clinical ramifications [9, 10].

Adenomyosis refers to pathological changes resulting in the thickening of the inner myometrium, while the endometrium is proliferative. The incidence of this disease in women at childbearing age ranges from 20 to 25% [11–13]. Over 50% of patients experience abnormal hemorrhage, pelvic pain, and fertility problems [5, 6, 14]. Adenomyosis is an occasionally chronic, immuno-inflammatory illness, involving multiple inflammatory factors, including TNF-α in the focal tissues, IL-1β, IL-6, and IL-8 [15]. A multicenter cohort study conducted in Japan reported that patients with diffuse adenomyosis had a greater prevalence of uterine infection; however, the study did not investigate whether these women had concurrent CE [16]. Through cross-sectional research, Khan et al. reported that the varying prevalence of CE in various types of adenomyosis could contribute to unfavorable reproductive outcomes [17]. Nevertheless, little data is available on the prevalence of CE in adenomyosis and patients who are infertile. This retrospective study aimed to explore the hysteroscopic characteristics of patients with infertility and adenomyosis combined with CE, and the correlation between CE and infertility in these patients.

## Materials and methods

### Ethical considerations

This study was approved by the ethics committee of our institution (No. KS-LS-2023-001). The need for informed consent was waived due to the retrospective nature of the study.

### Patients

We retrospectively reviewed 154 patients with infertility who underwent hysteroscopy procedures at Liuzhou Maternal and Child Health Care Hospital in Guangxi, China, between January 2020 and June 2023. Overall, 77 patients were diagnosed with adenomyosis, and the remaining 77 patients did not have adenomyosis, which was confirmed by histology or imaging.

The inclusion criteria included (i) age between 18 and 45 years; (ii) confirmed infertility (unable to conceive after a year of consistent sexual activity without using contraception); (iii) without a contraindication for surgery (e.g., serious cardiovascular disease, coagulopathy, or acute reproductive tract infection); and (iv) access to thorough medical information.

The exclusion criteria included: (i) history of antibiotic or anti-inflammatory medication use within 3 months before surgery; (ii) history of hysteroscopy within 3 months before surgery; (iii) history of intrauterine adhesions; (iv) history of uterine anomalies, submucous myomas, endometrial polyps, intrauterine hyperplasia, and endometrial tuberculosis; and (v) diagnosed malignant uterine tumor.

As reported previously [18], all patients with adenomyosis underwent 1.5T MRI for evaluation for at least 6 months before hysteroscopic surgery. MRI is considered the gold standard for the noninvasive identification of adenomyosis in patients with infertility [19] and can clearly demonstrate the presence of adenomyosis with either symmetric or asymmetric lesions in the internal or exterior layers of the myometrium [20]. The normal junctional zone (JZ) is located immediately beneath the endometrium and represents the innermost compact layer of the myometrium [21]. Patients with adenomyosis may exhibit focal or diffuse JZ hypertrophy. A JZ thickness of 12 mm or greater is the most frequently used threshold for diagnosis of adenomyosis, while a measurement of less than 8 mm has a high negative predictive value for the presence of the disorder [22, 23]. Based on the relationship between the adenomyotic lesion, uterine serosa, and endometrium, 77 patients with adenomyosis were classified into four subgroups: internal, exterior, intramural, and full-thickness.

Every surgical operation was performed under general anesthesia and by experienced gynecological surgeons. All the surgeries were performed in the follicular phase of the menstrual cycle using a rigid hysteroscope (KMS, Hunan, China). An advancing hysteroscope was used to carefully examine the front and back walls, two flank walls, two sides of the cervix, and cervical mucosa across endometrial surfaces throughout this assessment. This technique allowed for a close inspection of potential macroscopic indicators for CE, such as uterine cavity morphology, intima thickness, color, elasticity, smoothness, glands, stroma, and oviductal orifice [24]. Discrete or widespread micropolyps, stromal edema, and generalized periglandular hyperemia were the signs observed during hysteroscopy to identify CE [25–27]. An endometrial biopsy was performed at the end of the procedure. The formalin-fixed biopsy samples were sent to the Department of Pathology for a histological assessment. The presence of at least five plasma cells in the endometrial stroma per ten HPF confirmed a CE diagnosis following an immunohistochemistry (IHC) labeling using a CD138 antibody [9, 10].

### Statistical analysis

Statistical analysis and graphical representations were performed using SPSS 26.0 (SPSS Inc., Chicago, IL). Normal distribution data are displayed as mean ± SD, whereas skewed distribution data are displayed as median and interquartile range. To contrast different grouping variables, one-way analysis of variance or the Kruskal-Wallis test was performed. To ascertain the distinctions among subgroups, a post-hoc test was carried out when there was a significant difference. Count data are reported as percentages (%), and the chi-square test or Fisher’s exact test was carried out. Subgroup differences were confirmed using a post-hoc Bonferroni test when there was a significant difference. Univariate and multivariate logistic regression analyses were performed to examine the factors influencing CE. Statistical significance was set at *p* < .050.

## Results

### Clinical parameters

A total of 77 patients were assigned to the cohort with adenomyosis, while another 77 cases were assigned to the cohort without adenomyosis. Table 1 presents the research population’s demographic characteristics. No statistically significant differences in the length of infertility, infertility history, time of pregnancy, abortion, and delivery, anti-Müllerian hormone (AMH) levels, or the presence of hydrosalpinx were observed between the two groups. Considerable variations were observed in age at menarche, length of menstrual cycle, body mass index (BMI), and CA125 levels across both cohorts. In comparison to the non-adenomyosis group, the adenomyosis group had significantly higher BMI and CA125 levels, although the menstrual cycle was significantly shorter and the age at menarche was significantly lower (Table 1).

**Table 1:** Clinical characteristics of patients (AM and non-AM cohorts)

Variable	AM cohort(*n* = 77)	Non-AM cohort(*n* = 77)	*P* value
Age(years)	36.0(32.5, 39.0)	36.0(33.0, 39.5)	0.500
Age of menarche	12.0(11.0,12.0)	13.0(12.0,13.0)	0.000
menstrual cycle(days)	28.0(26.3,29.0)	29.0(28.0,30.0)	0.000
BMI (kg/m2)	26.8(24.7,30.4)	25.5(23.7,27.9)	0.017
Length of infertility(years)	4.0(3.0,5.5)	4.0(2.0,6.0)	0.339
Infertility			0.164
Primary infertility(%)	36.4%(28/77)	26.0%(20/77)	
Secondary infertility(%)	63.6%(49/77)	74.0%(57/77)	
Pregnancy(n)	1.0(0.0,3.0)	1.0(0.0,3.0)	0.453
Abortion(n)	1.0(0.0,3.0)	1.0(0.0,3.0)	0.562
Delivery(n)	0.0(0.0,1.0)	0.0(0.0,1.0)	0.185
AMH(ng/ml)	2.7(2.0,3.9)	2.7(2.2,3.7)	0.533
CA125(U/ml)	39.0(0.35.0,50.0)	15.0(9.5,20.0)	0.000
Presence of hydrosalpinx(%)	48.1%(37/77)	33.8%(26/77)	0.071

### Hysteroscopic features and CD138 immunohistochemical staining

Compared with the non-adenomyosis group, the adenomyosis group had a higher diagnostic rate of CE (75.3% vs. 46.8% according to hysteroscopy and 74.0% vs. 33.8% according to histopathology, both *p* < .050). Each hysteroscopic feature associated with CE was analyzed individually. In patients with adenomyosis, the primary manifestation linked to a hysteroscopic diagnosis of CE was the presence of micropolyps, while in patients without adenomyosis, it was hyperemia. Micropolyps were detected in 51.7% of cases in the adenomyosis cohort and 27.8% in the non-adenomyosis cohort, indicating a considerably higher incidence of micropolyps in the adenomyosis group (*p* < .050). The prevalence of edema, hyperplasia, or hyperemia did not significantly differ between the two cohorts (Table 2). Additionally, the incidence of CE in patients with internal adenomyosis was significantly lower than in the other three types, as determined by hysteroscopy or histopathology (Table 3).

**Table 2:** The diagnosis of CE based on hysteroscopy and histopathology (AM and non-AM cohorts)

Variable	AM cohort(*n* = 77)	Non- AM cohort(*n* = 77)	*P* value
Hysteroscopy(+)(%)	75.3%(58/77)	46.8%(36/77)	0.000
Hyperemic(%)	36.2%(21/58)	47.2%(17/36)	0.290
Micropolyps(%)	51.7%(30/58)	27.8%(10/36)	0.022
Edema hyperplasia(%)	34.5%(20/58)	38.9%(14/36)	0.667
CD138 IHC(+)(%)	74.0%(57/77)	33.8%(26/77)	0.000

**Table 3:** Comparison of incidence of CE in patients with different types of adenomyosis

Parameter	Internal(*n* = 25)	External(*n* = 17)	Intramural(*n* = 10)	Full-thickness(*n* = 25)	*P*
Hysteroscopy(+)(%)	56.0%(14/25)a	76.5%(13/17)	100%(10/10)	84.0%(21/25)	0.026
CD138 IHC(+)(%)	48.0%(12/25)a	82.4%(14/17)	90.0%(9/10)	88.0%(22/25)	0.006

Notes: a. Indicates a significant difference between patients with internal adenomyosis and patients with external, intramural, and full-thickness adenomyosis (*p* < .05).

**Table 4:** Univarlate and multivariate logistic regresslon analysis of factors influencing CE

Variable	Univariate logistic OR (95%CI)	*P*	Multivariate logistic OR (95%CI)	*P*
Age(years)	1.036	0.541	-	-
BMI (kg/m2)	1.594	0.000	1.535	0.007
AMH(ng/ml)	1.017	0.944	-	-
CA125(U/ml)	1.090	0.011	1.087	0.093
Pregnancy frequency	0.796	0.072	-	-
Abortion frequency	0.718	0.021	0.660	0.053
Presence of endometriosis	0.549	0.280	-	-
Presence of hydrosalpinx积水	0.847	0.751	-	-
History of uterine cavity surgery frequency	0.830	0.171	-	-
Type of adenomyosis (vs. Full-thickness)				
Internal	0.128	0.007	0.116	0.030
External	0.192	0.039	0.375	0.315
Intramural	0.635	0.643	0.149	0.126
Location of lesions(vs. Fundus)				
Anterior	1.038	0.954	-	-
Posterior	0.677	0.551	-	-

### Logistic regression analysis of variables affecting CE in patients with adenomyosis

Based on histopathology, the 77 patients with adenomyosis were divided into two groups: 20 without CE (non-CE group) and 57 with CE (CE group). The risk variables for CE were investigated using univariate logistic regression analysis. No significant correlation was observed between patient age, AMH level, pregnancy frequency, presence of endometriosis, presence of hydrosalpinx, history of uterine cavity surgery, or lesion number or location. However, notable distinctions were observed between the CE and non-CE groups regarding patient BMI, CA125 levels, frequency of abortions, and adenomyosis type (internal vs. full-thickness).

We evaluated the associations between BMI, CA125 levels, abortion frequency, adenomyosis type, and CE predisposing factors to validate the findings of the univariate logistic regression analysis. Compared to patients with internal adenomyosis, those with full-thickness adenomyosis had a significantly higher CE rate (Table 4).

**Fig. 1:**
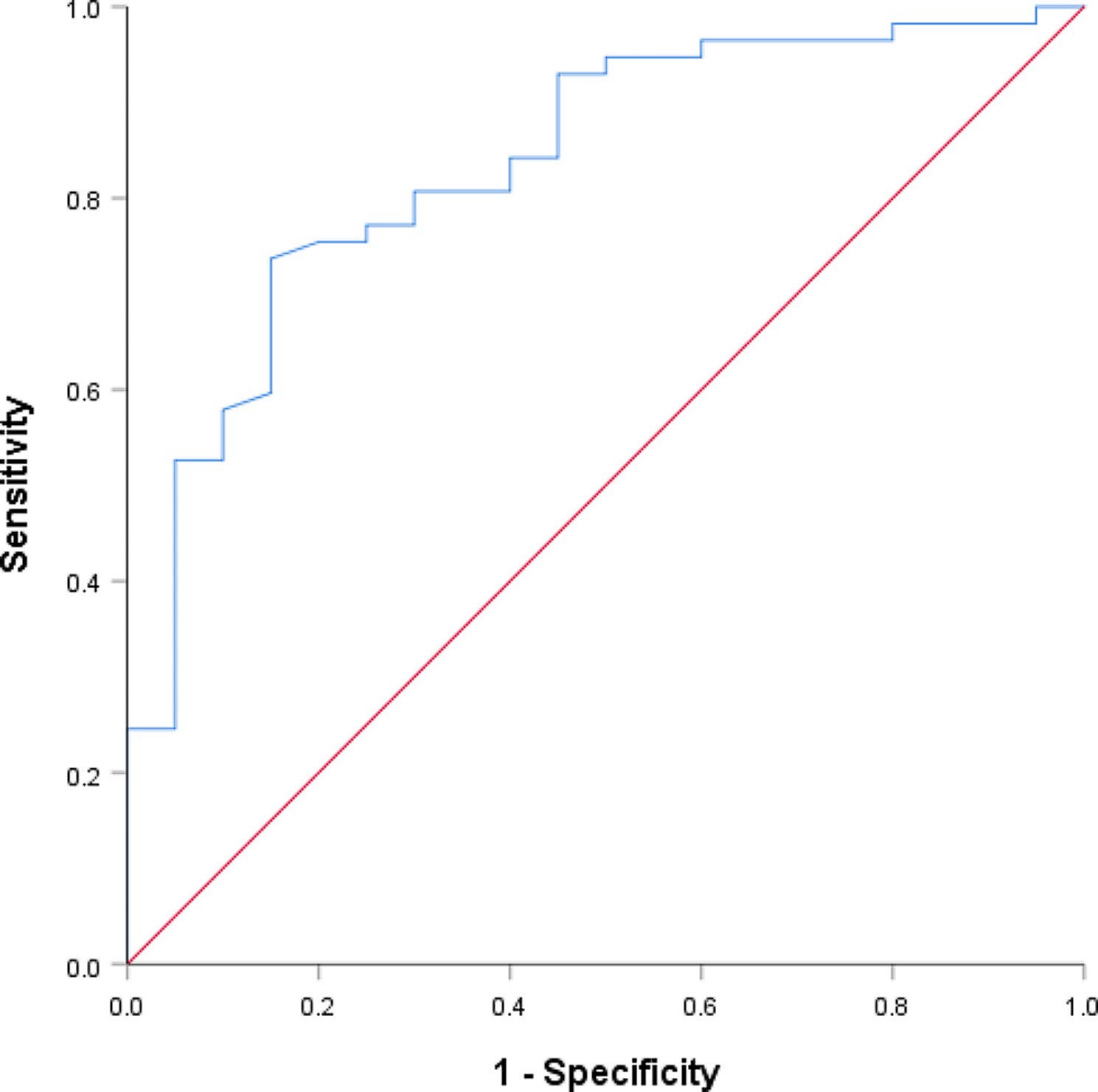
Receiver operating characteristic curves

Using receiver operating characteristic (ROC) curves, we examined the connection between BMI and CE rates. Based on the information, BMI had the highest Youden Index (26.07) for age. Our findings showed that patients’ CE rates were significantly higher in those with a BMI > 26.07 kg/m^2^ (Fig. 1).

## Discussion

This study compared the uterine cavity conditions of patients with and without adenomyosis and confirmed the presence of endometritis through histopathological and immunohistochemical analyses. Adenomyosis is being discovered in infertile women more frequently as a result of the current tendency of women delaying pregnancy until their late thirties or early forties [13]. Previous studies have suggested that multiple parity is a protective factor against adenomyosis, whereas a short menstruation cycle, early age of menarche, history of depression, and increased BMI are considered promoting factors [28–30]. Our study found that the BMI and CA125 levels were significantly higher in the adenomyosis cohort than in the non-adenomyosis cohort. However, the age at menarche was significantly lower, and the menstrual cycle was significantly shorter, which supports the findings of previous studies. In our opinion, early menarche can be interpreted as a surrogate indicator of high levels of pro-inflammatory estrogen exposure during development [31], and recurrent menstruation-induced impaired spontaneous decidualization could increase the likelihood of adenomyosis development.

The exact mechanism behind the correlation between infertility and adenomyosis remains elusive. Nevertheless, several hypotheses have been proposed to address this association, including altered endometrial function/receptivity, impairment of utero-tubal sperm transport, menstrual cycle disruption, local inflammation triggered by adenomyosis, and dysregulation of local hormone metabolism, culminating in a hyperestrogenic local environment [32]. The incidence of CE brought on by intrauterine microbial infections may be linked to poor reproductive outcomes in women with adenomyosis [17]. Several recent investigations have demonstrated that women with and without adenomyosis have distinct vaginal microbiota patterns [33]. Notably, adenomyosis is associated with a distinctive endometrial microbiota profile, with certain pathogenic bacteria such as *Citrobacter freundii*,* Prevotella copri*, and *Burkholderia cepacia* being implicated [34]. The activation of cytokines that promote inflammation, coupled with the production of virulence factors by specific endometrial microbiota, subsequently influences various cellular processes, including inflammation, immunoregulation, survival, proliferation, invasion, and angiogenesis of endometrial cells. [35–37]. Based on available data, the association between adenomyosis and poor reproductive outcomes may be attributed to the destruction of the microvillus and axonemal alteration within the apical endometrium due to endometrial inflammation [38]. Currently, CE diagnosis is based on the results of endometrial biopsy and hysteroscopic examination. Despite having limited participants, our study provides a deeper understanding of the prevalence of endometritis in patients with adenomyosis and infertility. Our findings demonstrated that compared to the non-adenomyosis group, the adenomyosis group had a significantly higher prevalence of CE. According to Khan et al., women with adenomyosis had a higher incidence of CE (about 60%) than those with uterine myomas (10%) [17]. In contrast, both cohort groups showed a higher prevalence of CE, confirming high prevalence of CE in low-fertility population. However, there was a lack of consensus between histological and hysteroscopic findings in each investigation (with agreement ranging from 46.5 to 95% for CE diagnosis or exclusion) (34). Even in instances where hysteroscopic indications of CE are present, confirmation through endometrial biopsy and histological analysis remains imperative. Histology remains the gold standard diagnostic approach owing to discrepancies frequently observed between hysteroscopy and histopathology outcomes among patients. Consequently, relying solely on positive hysteroscopic findings without histological verification may lead to erroneous CE diagnoses, potentially resulting in overdiagnosis [39].

Our results also showed that the main presentation of a hysteroscopic diagnosis of CE in patients with adenomyosis was micropolyps, whereas that in the non-adenomyosis group was hyperemia. Micropolyps are recently discovered tiny intrauterine growths < 1 mm in size and have a unique connective vascular axis that can be found across the endometrial surface or in specific locations [26]. The histological examination of the connective axis of micropolyps reveals the presence of inflammatory cell accumulation; furthermore, it is proposed that micropolyps signify an endometrial proliferative stimulus owing to an increased level of inflammation [26, 27]. It is speculated that the appearance of micropolyp features may be related to changes in endometrial receptors in patients with adenomyosis. Estrogen receptor expression increases with an increase in inflammation, whereas progesterone receptor expression decreases in the border area, inducing progesterone resistance [40], damaging endometrial health, and leading to local tissue edema and proliferation. However, the specific pathological and physiological changes require further exploration.

Based on the findings of previous studies, the different subtypes may represent heterogeneous etiologies and pathogenesis. Intrinsic adenomyosis may occur when the endometrium traverses directly into the inner and medial myometrium. External adenomyosis may occur as a consequence of direct invasion of the serosa by extrauterine endometriosis. Intramural adenomyosis may be caused by metaplasia or epithelial-mesenchymal transition (EMT). A diverse combination of different types of advanced disease may result in full-thickness adenomyosis [41]. Prior research [18] has established a strong correlation between variations in clinical manifestation and pregnancy outcomes and the classification of adenomyosis. Consequently, it is indisputable that diverse treatment approaches and subtype-specific adenomyosis analysis must be considered when analyzing the impact of adenomyosis on pregnancy outcomes. In this study, patients with internal adenomyosis had the lowest incidence of CE. Additionally, the incidence of CE was found to be influenced by the type of adenomyosis, as indicated by the multivariate logistic regression analysis. Compared with patients with internal adenomyosis, those with full-thickness adenomyosis had a significantly higher CE rate. As adenomyotic lesions progress and become stiffer due to increased fibrosis, pro-fibrotic molecules originating from the lesions migrate toward the adjacent endometrial-myometrial interface [42]. EMT and fibroblast-to-myofibroblast trans-differentiation are facilitated by macrophage-secreted inflammatory cytokines and growth factors, which ultimately result in lesion invasion and fibrosis [43]. The pain associated with adenomyosis was found to be positively correlated with the quantity of accumulated macrophages [44]. Based on our previous findings, patients with full-thickness adenomyosis experienced more menstrual discomfort than those with internal adenomyosis [18]. Combining the above mechanisms and clinical manifestations may help explain this phenomenon. Conversely, Khan et al. discovered that intrinsic adenomyosis had a considerably higher tissue infiltration of macrophages in the endometria than extrinsic adenomyosis. However, a lack of substantial differentiation in the incidence of CE was seen between the two adenomyosis types. Similarly, in endometria from women with various forms of adenomyosis, there were no appreciable variations in the location of CD138-stained plasma cells [17]. The endometrial regions and phases of the menstrual cycle of the samples may account for these variations. Epithelial cells derived from the secretory phase endometrium of adenomyotic uteri exhibited decreased levels of pro-inflammatory cytokines in vitro. This finding may indicate that stromal cells present in adenomyosis lesions, specifically during the proliferative phase, play a significant role in mediating the locally dysregulated immunological reaction [45]. Incidence of CE was found to be considerably higher on the homolateral side compared to the contralateral side in patients with localized adenomyosis [17]. All our research samples were in the proliferative phase, but there were no specific endometrial regions. This study offers an exhaustive foundation for subsequent investigations in this field.

Women with uncontrolled endometrial inflammation are prone to developing adenomyosis, the central component of uterine adenomyotic lesions. However, the causal relationship between adenomyosis and CE remains unclear, and a comprehensive assessment of its effects on female fertility is required. Using univariate and multivariate analyses, we investigated the factors that contribute to CE in patients with adenomyosis and infertility. Multiple logistic regression analysis findings indicated that BMI influences the incidence of CE in addition to adenomyosis type. One case-control study showed that overweight or obese women had a higher risk of developing adenomyosis [46]. Women diagnosed with adenomyosis were more prone to central obesity and had reduced levels of high-density lipoprotein C, according to recent research examining the individual components of metabolic syndrome [47]. In a study on polycystic ovary syndrome (PCOS), it was found that obesity increases both hyperandrogenism and low-grade inflammation in patients with PCOS [48]. The degree of obesity has a major impact on the reproductive system in women. Increased endometrial levels of free fatty acids can negatively impact endometrial function in obese PCOS patients [49]. Weight loss is a well-known element in restoring endometrial function [50]. Our study revealed a significantly higher CE incidence in patients with a BMI > 26.07 kg/m^2^ based on ROC curves, and we hypothesized that BMI may have a predictive role in the CE incidence rate in adenomyosis, and weight loss may reduce the incidence of adenomyosis and CE. However, more studies on metabolic risk factors related to adenomyosis must be conducted to confirm this hypothesis.

The design of this study had some limitations. First, the study examined patients’ observations from a single location and was restricted to certain types of samples. Future research should consider different menstrual cycle stages, and endometrial regions of the uterine cavity should be distinguished and detected during sampling. Second, hysteroscopy and endometrial biopsy were performed by different physicians, possibly introducing heterogeneity. Our current research has opened new directions for exploring the exact mechanism and causal relationship between CE and adenomyosis, as well as their impact on fertility outcomes. Further research using larger sample sizes and more comprehensive data is required to validate the correlation between CE and adenomyosis in patients with infertility.

## Conclusions

We retrospectively studied incidence of CE in patients with infertility with different forms of adenomyosis and attempted to explore the related high-risk factors. The prevalence of CE is significantly higher in patients with adenomyosis and infertility. The differences in the incidence of CE are closely associated with the classification of adenomyosis. When patients with infertility are diagnosed with adenomyosis, it is recommended to clarify the classification and screen for endometritis. Further research is required to explore the mechanisms underlying these effects.

## Data Availability

The datasets used and/or analysed during the current study available from the corresponding author on reasonable request.

## Abbreviations

CE: chronic endometritis
MRI: magnetic resonance imaging
BMI: body mass index
RPL: recurrent pregnancy loss
RIF: repeated implantation failure
HPF: High-power field
IHC: immunohistochemistry
AMH: anti-Müllerian hormone
ROC: receiver operating characteristic
EMT: epithelial-mesenchymal transition
PCOS: polycystic ovary syndrome
